# Diversity of Marine 1,3-Xylan-Utilizing Bacteria and Characters of Their Extracellular 1,3-Xylanases

**DOI:** 10.3389/fmicb.2021.721422

**Published:** 2021-10-01

**Authors:** Hai-Ning Sun, Chun-Mei Yu, Hui-Hui Fu, Peng Wang, Zai-Guang Fang, Yu-Zhong Zhang, Xiu-Lan Chen, Fang Zhao

**Affiliations:** ^1^State Key Laboratory of Microbial Technology, Marine Biotechnology Research Center, Shandong University, Qingdao, China; ^2^College of Marine Life Sciences, Frontiers Science Center for Deep Ocean Multispheres and Earth System, Ocean University of China, Qingdao, China; ^3^Laboratory for Marine Biology and Biotechnology, Pilot National Laboratory for Marine Science and Technology, Qingdao, China; ^4^Key Laboratory of Tropical Biological Resources of Ministry of Education, College of Marine Science, Hainan University, Haikou, China

**Keywords:** marine bacteria, diversity, marine algae, 1,3-xylan, extracellular 1,3-xylanases

## Abstract

1,3-xylan is present in the cell walls of some red and green algae and is an important organic carbon in the ocean. However, information on its bacterial degradation is quite limited. Here, after enrichment with 1,3-xylan, the diversity of bacteria recovered from marine algae collected in Hainan, China, was analyzed with both the 16S rRNA gene amplicon sequencing and the culture-dependent method. Bacteria recovered were affiliated with more than 19 families mainly in phyla *Proteobacteria* and *Bacteroidetes*, suggesting a high bacterial diversity. Moreover, 12 strains with high 1,3-xylanase-secreting ability from genera *Vibrio*, *Neiella*, *Alteromonas*, and *Gilvimarinus* were isolated from the enrichment culture. The extracellular 1,3-xylanases secreted by *Vibrio* sp. EA2, *Neiella* sp. GA3, *Alteromonas* sp. CA13-2, and *Gilvimarinus* sp. HA3-2, which were taken as representatives due to their efficient utilization of 1,3-xylan for growth, were further characterized. The extracellular 1,3-xylanases secreted by these strains showed the highest activity at pH 6.0–7.0 and 30–40°C in 0–0.5M NaCl, exhibiting thermo-unstable and alkali-resistant characters. Their degradation products on 1,3-xylan were mainly 1,3-xylobiose and 1,3-xylotriose. This study reveals the diversity of marine bacteria involved in the degradation and utilization of 1,3-xylan, helpful in our understanding of the recycling of 1,3-xylan driven by bacteria in the ocean and the discovery of novel 1,3-xylanases.

## Introduction

Marine algae generate approximately 104.9 petagram organic carbon per year that mainly deposit as polysaccharides, the most promising candidates for biomass conversion (Field et al., 1998; Hehemann et al., 2014). There are many unusual polysaccharides in the ocean that are not found in land plants, including 1,3-xylan. In the cell walls of land plants, xylans are the most abundant polysaccharides secondary to cellulose. They are heteroxylans, and their β-1,4-linked D-xylopyranosyl backbone can be replaced by various side chains, such as α-L-arabinose, 4-O-methyl-glucuronic acid, and acetate (Déjean et al., 2013). Different from that, 1,3-xylan is a linear homopolysaccharide composed of β-1,3-linked D-xylose units (Iriki et al., 1960). 1,3-xylan, which is considered the main xylan structure in marine algae (Qeshmi et al., 2020), has been found in the cell walls of many green algae (*Caulerpa*, *Dichotomosiphon*, *Halimeda*, *Penicillus*, and *Udotea* spp.) and some red algae (*Porphyra* and *Bangia* spp.; Iriki et al., 1960; Veluraja and Atkins, 1987; Usov and Zelinsky, 2013).

1,3-xylanases (EC 3.2.1.32), capable of cleaving the β-1,3-xylosidic linkages in 1,3-xylan, are crucial in the degradation and recycling of 1,3-xylan in the ocean. They also represent a vast potential for algal biomass conversion, functional xylooligosaccharides production, and protoplast preparation (Araki et al., 1994; Maeda et al., 2012; Umemoto et al., 2012). However, to date, only a limited number of 1,3-xylanases have been studied. In the 1980s, six extracellular 1,3-xylanases produced by a fungus, *Aspergillus terreus* A-07, were purified (Chen et al., 1986). Later, several 1,3-xylanase-secreting marine bacteria were isolated, which are *Pseudomonas* sp. PT-5 (Yamaura et al., 1990), *Alcaligenes* sp. XY-234 (Araki et al., 1998), *Vibrio* sp. XY-214 (Araki et al., 1999), and *Vibrio* sp. AX-4 (Araki et al., 1987; Kiyohara et al., 2005). The genes encoding 1,3-xylanases from these bacteria were cloned and functionally characterized. In recent years, three other 1,3-xylanases were experimentally identified from *Thermotoga neapolitana* DSM 4359 (Okazaki et al., 2013), *Pseudomonas vesicularis* MA103 (Liang et al., 2015), and *Flammeovirga pacifica* WPAGA1 (Cai et al., 2018). So far, all the discovered 1,3-xylanases belong to glycoside hydrolase (GH) family 26 according to the assignment in the Carbohydrate Active Enzymes (CAZy) database (Lombard et al., 2014).^1^ Despite these studies, bacterial degradation on 1,3-xylan is still a largely unexplored field. The diversity of marine bacteria participating in the degradation and utilization of 1,3-xylan still lacks investigation, and more 1,3-xylanase-secreting bacteria and 1,3-xylanases await discovery and exploitation.

Algal surfaces harbor a rich community mainly composed of bacteria that can benefit from various organic substances produced by algae (Armstrong et al., 2001; Lachnit et al., 2011). They are good sources to isolate bacteria capable of degrading algal polysaccharides (Dong et al., 2012; Martin et al., 2015). In this study, we investigated the diversity of 1,3-xylan-utilizing bacteria associated with marine algae *Caulerpa* sp. and *Chaetomorpha* sp. and characterized the extracellular 1,3-xylanases secreted by several representative strains. Algal samples were collected from a *Caulerpa lentillifera* aquaculture base and the nearby beach in Hainan, China. After enrichment with 1,3-xylan, the bacterial diversity was analyzed using the 16S rRNA gene amplicon sequencing. The diversity of culturable bacteria was also analyzed. Moreover, 12 strains with high 1,3-xylanase-secreting ability belonging to 4 genera were isolated and identified. The extracellular 1,3-xylanases secreted by 4 representative strains were further characterized. The results shed new light on 1,3-xylan degradation and 1,3-xylanase-secreting bacteria.

## Materials and Methods

### Sample Collection

**Figure 1:**
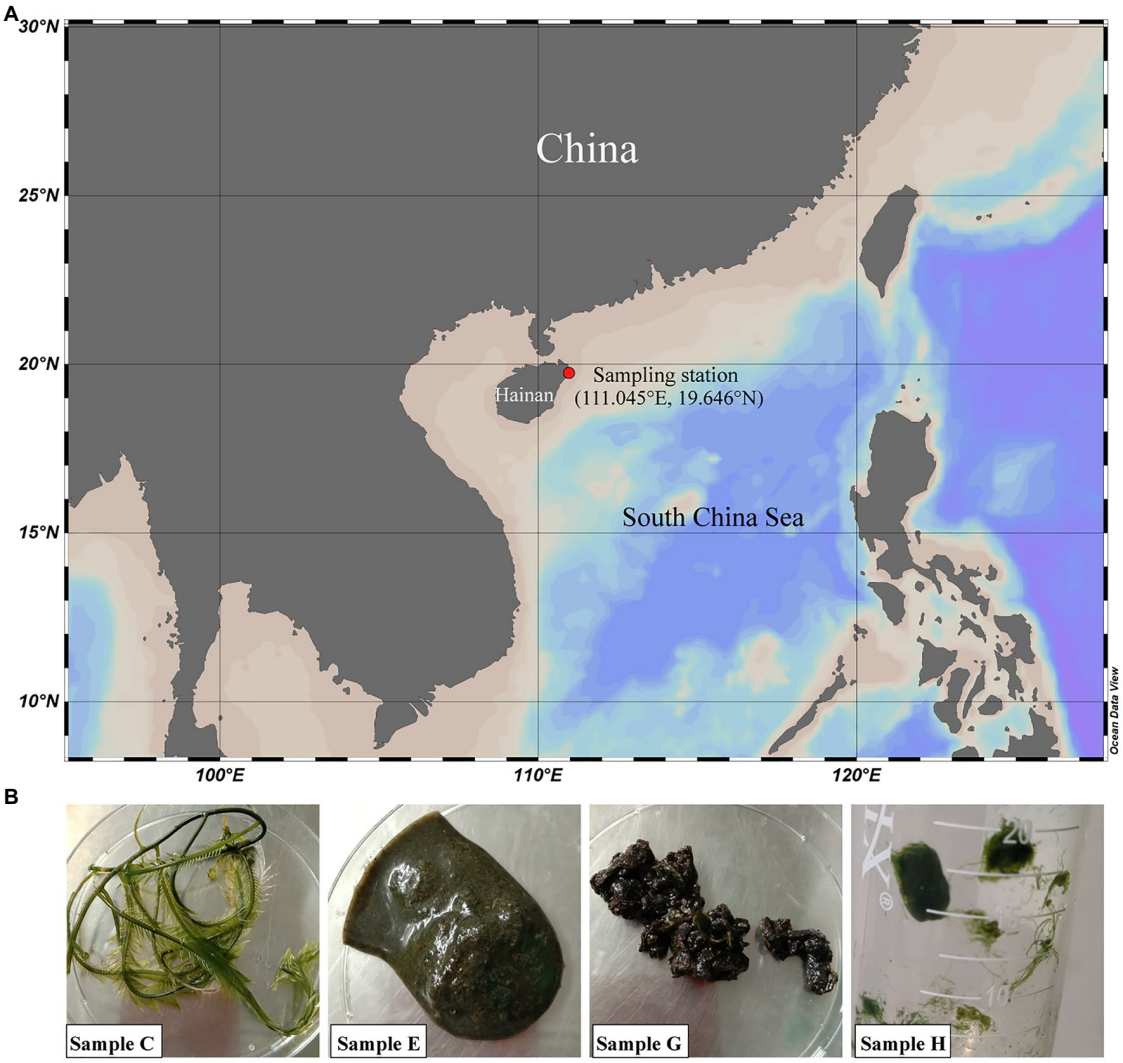
Geographic location of the sampling station **(A)** and sample images **(B)**. At the sampling station, fresh and rotten seaweeds were collected from a *Caulerpa lentillifera* aquaculture base (samples C, E, and H) and the nearby beach (sample G) in Wenchang, Hainan, China. Samples C, E, G, and H are *Caulerpa sertularioides*, rotten *Caulerpa lentillifera*, unknown rotten algae and *Chaetomorpha* sp., respectively.

Four algal samples, named C, E, G, and H, were collected from a *Caulerpa lentillifera* aquaculture base and the nearby beach in Wenchang, Hainan Province, China (111.045°E, 19.646°N) in September 2019 (Figure 1A). Samples C, E, and H were from the *Caulerpa lentillifera* aquaculture base, and sample G was from the beach. Samples C, E, G, and H are *Caulerpa sertularioides*, rotten *Caulerpa lentillifera*, rotten algae of which the taxonomy was unable to determine and *Chaetomorpha* sp., respectively (Figure 1B). The temperature and pH of the seawater in the sampling sites were 30°C and 8.0–8.2, respectively.

### 1,3-Xylan and 1,3-Xylooligosaccharides Preparation

1,3-xylan was extracted from *Caulerpa lentillifera* according to the method of Lahaye et al. (2003) with minor modifications. Briefly, *Caulerpa lentillifera* was washed with deionized water, kiln-dried, and smashed into a powder. Then, the algal powder (10g) was boiled in 1.25% NaOH (500mL) for 30min. The same operation was repeated with 1.25% H_2_SO_4_, and then, the sample was bleached with 1.0% NaClO_4_ (500mL) at room temperature. Subsequently, 1,3-xylan was extracted with 10% NaOH (500mL) at 4°C for 4h and precipitated with absolute ethanol (2L) at 4°C overnight. The precipitate was collected and washed with 33% acetic acid and deionized water. Finally, the precipitate was freeze-dried to obtain water-insoluble 1,3-xylan. 1,3-xylooligosaccharides were prepared by enzymatic hydrolysis of 1,3-xylan with 1,3-xylanase XYL4 from *Vibrio* sp. AX-4 as previously described (Kiyohara et al., 2006). The gene encoding XYL4 was synthesized in the Beijing Genomics Institute (BGI; Beijing, China) and expressed in *Escherichia coli* BL21(DE3). The recombinant XYL4 was purified with Ni^2+^-nitrilotriacetic acid (NTA) resin (GE Healthcare, USA) followed by desalination on PD-10 desalting columns (GE Healthcare, USA). The purified XYL4 was stored in 10mM Tris–HCl buffer (pH 8.0) containing 100mM NaCl at −80°C for further use. Enzymatic hydrolysis of 1,3-xylan by the purified XYL4 to prepare 1,3-xylooligosaccharides was carried out as follows: A reaction mixture consisting of 0.4g 1,3-xylan and 200mg XYL4 in 40mL phosphate-buffered saline (PBS; 20mM, pH 7.0) was incubated at 30°C for 24h. The products were then separated by gel filtration on a Superdex 30 Increase 10/300 GL column (GE Healthcare, USA) by high-performance liquid chromatography (HPLC) equipped with a RID-20A refractive index detector (HPLC-RID). The injected volume was 100μL. The products were eluted with deionized water for 60min with a flow rate of 0.37mL/min. A mixture of xylose and 1,4-xylooligosaccharides (1,4X2-X6; Megazyme, Ireland) was used as the marker. The peaks corresponding to 1,3-xylobiose (1,3X2) and 1,3-xylotriose (1,3X3) were collected for further use (Supplementary Figure 1).

### Enrichment Cultivation With 1,3-Xylan

Two media, A and B, were used for enrichment cultivation. Medium A contained 0.2% 1,3-xylan, 0.05% NH_4_Cl, and 0.1% (v/v) vitamin solution in artificial seawater (ASW). Medium B contained the same components as medium A and an additional organic nitrogen source, 0.02% casein hydrolysate. ASW was composed of 30g NaCl, 6g Tris, 3g MgCl_2_^.^6H_2_O, 2g K_2_SO_4_, 0.2g K_2_HPO_4_, 10mg CaCl_2_, 6mg FeCl_3_^.^6H_2_O, 5mg Na_2_MoO_2_^.^7H_2_O, and 4mg CuCl_2_^.^2H_2_O in 1L deionized water (Li et al., 2021). The pH was adjusted to 8.0 with HCl to keep consistence with that of the sampling sites (8.0–8.2). The compositions of the vitamin solution were 20mg nicotinic acid, 20mg pyridoxine-HCl, 20mg riboflavin, 20mg calcium pantothenate, 10mg thiamine-HCl, 10mg p-aminobenzoic acid, 1mg biotin, and 1mg cyanocobalamin in 1L deionized water (Kanagawa et al., 1982). Each algal sample was cut into small pieces using sterile scissors, and then, approximately 1g of each algal sample was put in 20mL media A and B. After aerobic incubation at 30°C with continuous shaking of 180rpm for 6–7days, 0.2mL enrichment culture was inoculated into 20mL the same fresh medium and incubated under the same conditions. After repeated enrichment, the bacterial diversity of each sample was analyzed in Shanghai Biozeron Biotechnology Co., Ltd. (Shanghai, China) by using the 16S rRNA gene amplicon sequencing. Briefly, the V3 to V4 hypervariable region of the bacterial 16S rRNA gene was amplified from the total bacterial genomic DNA using the universal primers 341F (5'-CCTAYGGGRBGCASCAG-3') and 806R (5'-GGACTACNNGGGTATCTAAT-3'). High-throughput sequencing of the PCR amplification products was performed on an Illumina MiSeq platform. The paired-end reads were merged into longer contigs by FLASH and quality-filtered by Trimmomatic. After removing chimeric sequences, the remaining sequences were assigned into operational taxonomic units (OTUs) at similarities of 97% using UPARSE version 7.1 (Edgar, 2013). The taxonomy of each 16S rRNA gene sequence was analyzed by the ribosomal database project (RDP) classifier algorithm against the Silva 16S rRNA database using a confidence threshold of 70% (Wang et al., 2007). The richness (Chao-1) and diversity (Shannon) indexes were analyzed using Mothur (Chappidi et al., 2019). Principal component analysis (PCA) was performed in R package Vegan and used to evaluate the differences among bacterial communities. Differences were considered significant at *p*-values less than 0.001.

### Culturable Bacteria Isolation

The enrichment cultures were diluted (10^−2^–10^−6^ dilution) and spread on the corresponding screening plates containing medium A or B supplemented with 1.5% agar powder. The plates were then incubated at 30°C until detectable colonies and hydrolytic zones formed. Morphologically different colonies were selected and purified with 2216E medium. The compositions of 2216E medium were 5g tryptone, 1g yeast extract, and 30g sea salts (Sigma, USA) in 1L deionized water. Strains with apparent hydrolytic zones were taken as 1,3-xylanase-secreting bacteria.

### Amplification of 16S rRNA Genes and Phylogenetic Analysis

The 16S rRNA genes were amplified by PCR with the primers 27F (5'-AGAGTTTGATCCTGGCTCAG-3') and 1492R (5'-GGTTACCTTGTTACGACTT-3'; Dong et al., 2012). The PCR products were sequenced in BGI (Beijing, China). The sequence alignment was performed in the EZBioCloud database^2^ (Yoon et al., 2017). The phylogenetic tree was constructed based on the neighbor-Joining method and using the Kimura 2-parameter model (Kimura, 1980) with MEGA X (Kumar et al., 2018).

### Growth Experiments and Extracellular 1,3-Xylanase Activity Assays

The strains *Vibrio* sp. EA2, *Neiella* sp. GA3, *Alteromonas* sp. CA13-2, and *Gilvimarinus* sp. HA3-2, which were all isolated from medium A, were aerobically cultured in 2216E medium at 30°C and 180rpm for 24h. The cells were collected, washed three times with sterile ASW, and resuspended in ASW to an optical density at 600nm of 0.8. Then, 1mL cell suspension was inoculated into 20mL medium A in triplicate and aerobically cultured at 30°C and 180rpm. At intervals of 6h, 1mL culture was removed into a 1.5-mL Eppendorf tube and let stand for 10min. As a result, water-insoluble 1,3-xylan precipitated and the bacterial cells still suspended in the culture. Then, the cell suspension was removed into a 96-well microplate and its optical density at 600nm was measured to generate growth curves. In the growth assays with *Caulerpa lentillifera*, the strains were cultured in the same conditions except that 1.0% dry *Caulerpa lentillifera* was used instead of 0.2% 1,3-xylan.

To determine the extracellular 1,3-xylanase activity, the culture (1mL) taken at intervals of 6h was centrifuged at 13,000rpm and 4°C for 5min, and the culture supernatant was collected. The extracellular 1,3-xylanase activity in the supernatant was determined by the dinitrosalicylic acid (DNS) method (Miller et al., 1960). A standard reaction system contained 10μL culture supernatant and 90μL 1,3-xylan (1.0%) in PBS (20mM, pH 7.0). The reaction system was incubated at 30°C for 1h (for the 1,3-xylanases from *Vibrio* sp. EA2 and *Alteromonas* sp. CA13-2) or 3.5h (for the 1,3-xylanases from *Neiella* sp. GA3 and *Gilvimarinus* sp. HA3-2). Then, the reaction was terminated by the addition of 200μL DNS, and the reaction mixture was boiled for 5min for coloring. Finally, the absorbance of the reaction mixture at 550nm was measured. One unit of enzyme activity is defined as the amount of enzyme required to release 1μmol xylose per min.

### Biochemical Characterization of Extracellular 1,3-Xylanases

*Vibrio* sp. EA2, *Neiella* sp. GA3, *Alteromonas* sp. CA13-2, and *Gilvimarinus* sp. HA3-2 were aerobically cultured in medium A at 30°C and 180rpm for 24h, 24h, 72h, and 30h, respectively, to their late-log phase. The cultures were centrifuged, and the supernatants were collected and used for the characterization of the extracellular 1,3-xylanases. The effect of temperature on the activity of extracellular 1,3-xylanases was determined from 10°C to 60°C in PBS at their respective optimum PHs. The effect of pH on the activity of extracellular 1,3-xylanases was determined with the Britton-Robinson buffer from pH 4.0 to 10.0 at their respective optimum temperatures. The effect of NaCl concentration on the activity of extracellular 1,3-xylanases was determined in PBS containing 0 to 5.0M NaCl at their respective optimum temperatures and pHs. In the thermo-stability assay, after incubation of 1,3-xylanases at 10°C to 60°C for different time periods, the residual activity was determined at their respective optimum temperatures and pHs in PBS. In the pH stability assay, 40μL 1,3-xylanases were diluted in 360μL Britton-Robinson buffers (pH 4.0–10.0) and preincubated at 4°C for 24h. After incubation, 3.6mg 1,3-xylan was added to the 400μL mixture and the residual activity was determined at the optimum temperature. 1,3-xylanases diluted in the corresponding Britton-Robinson buffers without preincubation were used as controls. Each experiment was performed in triplicate independently.

### HPLC Analysis of the Products Released From 1,3-Xylan by Extracellular 1,3-Xylanases

Culture supernatant from each strain (20μL) was incubated with 1.0% 1,3-xylan (180μL) in PBS (pH 7.0) for 24h. Then, the products were analyzed by HPLC equipped with an evaporative light scattering detector (HPLC-ELSD). The column used was a Superdex 30 Increase 10/300 GL column, and the eluent was deionized water. The injected volume was 30μL. The products were eluted with deionized water for 60min with a flow rate of 0.37mL/min. A mixture of xylose, 1,3X2, and 1,3X3 was used as the marker. The reaction system without 1,3-xylanases was used as the control.

### Nucleotide Sequence Accession Numbers

The 16S rRNA gene sequences of 12 1,3-xylanase-secreting bacteria in this study were deposited in GenBank with the following accession numbers: *Vibrio* sp. CB1, MZ340577; *Vibrio* sp. EA6, MZ340626; *Vibrio* sp. EA17, MZ342596; *Vibrio* sp. EA2, MZ342745; *Vibrio* sp. EA3, MZ342757; *Neiella* sp. GA1-2, MZ342758; *Neiella* sp. GA3, MZ342759; *Alteromonas* sp. CA11-2, MZ340547; *Alteromonas* sp. CA13-2, MZ340550; *Gilvimarinus* sp. HB14, MZ342767; *Gilvimarinus* sp. HA9-2S, MZ342768; and *Gilvimarinus* sp. HA3-2, MZ342787. The accession number for *Marisediminitalea* sp. HA8 is MZ821015, and the other 133 strains are from MZ816037 to MZ816169. The 16S rRNA gene amplicon sequencing data have been deposited in Sequence Read Archive (SRA) under the accession numbers from SRR15511288 to SRR15511295.

## Results

### Sample Description

1,3-xylan-containing green algae, including *Caulerpa lentillifera*, are widely distributed in tropical seas. To investigate the bacteria involved in 1,3-xylan degradation, a *Caulerpa lentillifera* aquaculture base and the nearby beach in Wenchang, Hainan Province, China, were chosen for sample collection (Figure 1A). Four algal samples were collected, named C, E, G, and H (Figure 1B), which are *Caulerpa sertularioides* (C), rotten *Caulerpa lentillifera* (E), rotten algae of which the taxonomy was unable to determine (G), and *Chaetomorpha* sp. (H), respectively (Figure 1B). Seawater in the sampling sites exhibited a slightly alkaline pH (8.0–8.2), and the temperature was approximately 30°C.

### Bacterial Diversity in Enrichment Media Analyzed With the 16S rRNA Gene Amplicon Sequencing

1,3-xylan, which was extracted from *Caulerpa lentillifera*, was used as the carbon source in media A and B to recover 1,3-xylan-utilizing bacteria from the 4 algal samples. Medium A contained NH_4_Cl as the nitrogen source. Medium B also contained an additional organic nitrogen source, casein hydrolysate, in case some 1,3-xylan-utilizing bacteria are not able to utilize inorganic nitrogen. The community compositions of the recovered bacteria were analyzed with the 16S rRNA gene amplicon sequencing. Based on the observed OTUs and Chao1 index, sample H showed the highest species richness and sample C showed the lowest species richness (Table 1). Between media A and B, the OTUs and Chao1 indexes of each sample were similar, indicating that the species richness of these samples was little affected by the nitrogen source (Table 1). According to the Shannon diversity indexes, in media A and B, sample G was the most diverse with the highest Shannon index and sample E was the least diverse with the lowest Shannon index (Table 1). Except sample H that exhibited a lower Shannon index in medium B than in medium A, the Shannon indexes were similar between media A and B for samples C, E, and G (Table 1), indicating that the nitrogen source generally had little influence on the bacterial diversity of the samples.

**Table 1:** Richness and diversity indexes of the bacteria recovered from algal samples with 1,3-xylan based on 16S rRNA gene amplicon sequencing.

Isolation sample	Medium A			Medium B		
	OTU observed	Chao1^a^	Shannon^a^	OTU observed	Chao1^a^	Shannon^a^
C	170	190 (178, 220)	2.43 (2.42, 2.44)	200	226 (211, 258)	2.70 (2.68, 2.71)
E	223	242 (231, 268)	1.61 (1.60, 1.63)	191	239 (215, 286)	1.65 (1.64, 1.67)
G	238	250 (243, 268)	3.61 (3.60, 3.62)	243	289 (264, 345)	3.36 (3.34, 3.37)
H	275	297 (285, 323)	3.16 (3.15, 3.18)	264	296 (279, 330)	2.61 (2.6, 2.63)

a Values in parentheses are lower bound of confidence interval (left) and upper bound of confidence interval (right).

**Figure 2:**
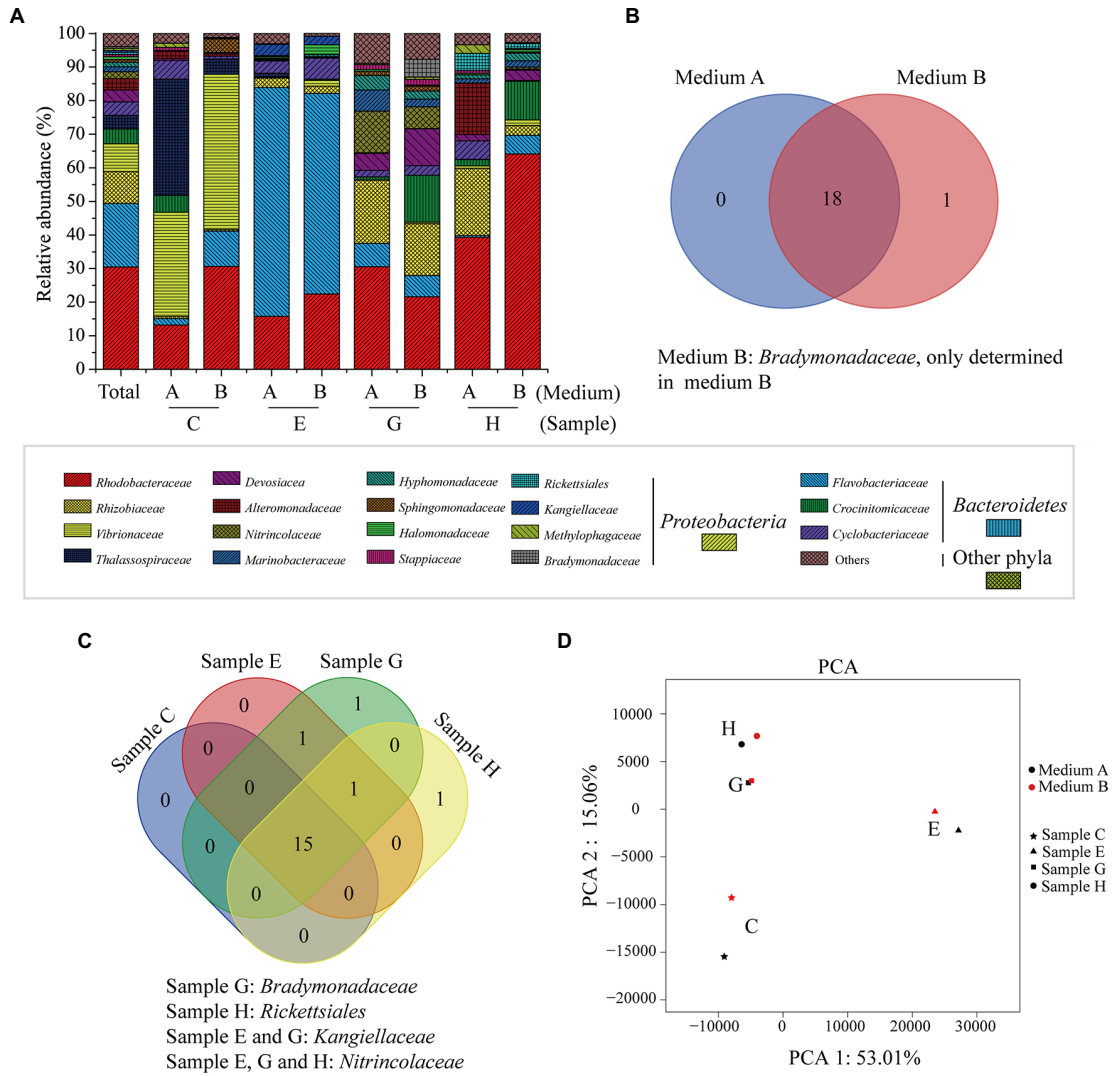
The bacterial communities recovered from algal samples with 1,3-xylan based on 16S rRNA gene amplicon sequencing. **(A)** Relative abundance of recovered bacteria at the family level. **(B)** A venn diagram analysis of recovered families from algal samples. **(C)** A venn diagram analysis of recovered families from media A and B. **(D)** Principal component analysis (PCA) of the bacterial communities.

Totally, 9 phyla, 15 classes, 55 orders, 94 families, and 194 genera were identified in these samples. At the phylum level, *Proteobacteria* (71.4%) and *Bacteroidetes* (28.1%) were two abundant groups, and the others accounted for less than 1% (Supplementary Figure 2). At the family level, there were 19 families with a relative abundance of higher than 1% in at least one sample, 18 of which were recovered from both media A and B (Figures 2A,B). Overall, *Rhodobacteraceae* (30.5%), *Flavobacteriaceae* (18.9%), *Rhizobiaceae* (9.5%), and *Vibrionaceae* (8.3%) were four abundant families (Figure 2A). Based on a Venn diagram analysis, 15 of the 19 families were common in all algal samples (Figure 2C), but the dominant families varied among these samples (Figure 2A; Supplementary Table 1). *Bradymonadaceae,* which was only recovered from medium B (Figure 2B), was unique in sample G, and *Rickettsiales* was unique in sample H (Figure 2C). The results indicated a diversity of the core bacteria involving in the 1,3-xylan utilization. The PCA result showed that the community structures of samples G and H were similar, which were significantly different from those of samples C and E (Figure 2D). In addition, the communities from media A and B exhibited only a slight difference for each sample, indicating that the nitrogen source had little impact on the community structure (Figure 2D), although the dominant families from media A and B varied for samples C, G, and H (Figure 2A; Supplementary Table 1). Together, these results reveal the high diversity of 1,3-xylan-utilizing bacteria associated with marine algae.

### Isolation and Diversity Analysis of Culturable Bacteria

After enrichment, bacteria were further isolated from the enrichment culture on plates with 1,3-xylan as the carbon source. After incubation at 30°C for 4–6days, a lot of colonies appeared on the plates. Morphologically different colonies (12–24 colonies each sample) were purified and identified. Totally, 146 strains were isolated, which were mainly distributed in *Proteobacteria* (87.7%) and *Bacteroidetes* (11.6%; Table 2). These strains were affiliated with 18 families in *Proteobacteria*, 5 families in *Bacteroidetes,* and 1 family in *Actinobacteria* (Table 2). Among these families, *Vibrionaceae* (16.4%) was predominant, followed by *Rhodobacteraceae* (13.0%), *Phyllobacteriaceae* (11.6%), and *Cellvibrionaceae* (9.6%; Table 2). A total of 33 genera were isolated, among which, 1, 6, 9, and 3 genera were exclusive to samples C, E, G, and H, respectively. *Vibrio* species were isolated from all algal samples (Table 2; Supplementary Figure 3). In addition, among the isolates, strains HA8 and HB14 share low 16S rRNA gene sequence similarities with *Marisediminitalea aggregate* WH169 (similarity: 95.13%) and *Gilvimarinus chinensis* DSM 19667 (similarity: 96.65%), respectively. They might be potential novel species and deserved to be investigated in the future. Together, these results further indicate a high diversity of marine bacteria capable of utilizing 1,3-xylan.

**Table 2:** Phylogenetic classification and relative abundance of culturable bacteria isolated from the enrichment cultures of the algal samples^a^.

Phylogenetic classification			Sample							
			C		E		G		H	
			Medium							
Phylum (No./Abundance)	Family (No./Abundance)	Genus (No.)	A	B	A	B	A	B	A	B
*Proteobacteria* (128/87.7%)	*Vibrionaceae* (24/16.4%)	*Vibrio* (24)	6	6	5	0	1	0	0	6
	*Rhodobacteraceae* (19/13.0%)	*Pseudoruegeri*a (8)	0	7	1	0	0	0	0	0
		*Ruegeria* (6)	0	0	1	2	0	0	3	0
		*Salipiger* (1)	0	0	0	0	1	0	0	0
		*Pseudooceanicola* (2)	0	1	0	0	0	1	0	0
		*Celeribacter* (2)	0	0	0	0	0	2	0	0
	*Phyllobacteriaceae* (17/11.6%)	*Nitratireductor* (16)	0	0	4	2	1	3	6	0
		*Mesorhizobium* (1)	0	0	0	1	0	0	0	0
	*Cellvibrionaceae* (14/9.6%)	*Gilvimarinus* (14)	0	0	4	0	0	0	4	6
	*Alteromonadaceae* (9/6.2%)	*Alteromonas* (7)	2	2	0	0	0	0	3	0
		*Marisediminitalea* (1)	0	0	0	0	0	0	1	0
		*Ningiella* (1)	0	0	0	0	0	1	0	0
	*Alteromonadales* (9/6.2%)	*Neiella* (9)	0	0	0	0	9	0	0	0
	*Pseudoalteromonadaceae* (7/4.8%)	*Pseudoalteromonas* (7)	1	0	0	0	4	0	1	1
	*Enterobacteriaceae* (5/3.4%)	*Enterobacter* (5)	0	2	0	1	0	2	0	0
	*Pseudomonadaceae* (5/3.4%)	*Pseudomonas* (5)	0	0	0	0	0	4	1	0
	*Marinobacteraceae* (5/3.4%)	*Marinobacter* (5)	0	0	1	0	0	0	0	4
	*Halomonadaceae* (4/2.7%)	*Halomonas* (4)	0	0	0	4	0	0	0	0
	*Thalassospiraceae* (3/2.1%)	*Thalassospira* (3)	2	0	0	0	0	0	1	0
	*Idiomarinaceae* (2/1.3%)	*Pseudidiomarina* (2)	0	0	0	0	1	1	0	0
	*Stappiaceae* (1/0.7%)	*Roseibium* (1)	1	0	0	0	0	0	0	0
	*Kangiellaceae* (1/0.7%)	*Kangiella* (1)	0	0	0	1	0	0	0	0
	*Erythrobacteraceae* (1/0.7%)	*Erythrobacter* (1)	0	0	0	0	0	1	0	0
	*Oceanospirillaceae* (1/0.7%)	*Marinobacterium* (1)	0	0	0	0	0	0	0	1
	*Hyphomonadaceae* (1/0.7%)	*Maricaulis* (1)	0	0	0	0	0	0	1	0
*Bacteroidetes* (17/11.6%)	*Crocinitomicaceae* (6/4.1%)	*Wandonia* (6)	0	0	0	0	0	3	2	1
	*Cyclobacteriaceae* (5/3.4%)	*Algoriphagus* (3)	0	0	1	2	0	0	0	0
		*Cyclobacterium* (2)	0	0	0	0	2	0	0	0
	*Flavobacteriaceae* (3/2.1%)	*Tenacibaculum* (2)	0	0	2	0	0	0	0	0
		*Mesonia* (1)	0	0	0	0	1	0	0	0
	*Prolixibacteraceae* (1/0.7%)	*Sunxiuqinia* (1)	0	0	0	0	0	1	0	0
	*Roseivirgaceae* (2/1.3%)	*Roseivirga* (2)	0	0	0	0	0	1	1	0
*Actinobacteria* (1/0.7%)	*Microbacteriaceae* (1/0.7%)	*Microbacterium* (1)	0	0	0	1	0	0	0	0
Total No.		146	12	18	19	14	20	20	24	19

a Strain names are shown in Supplementary Table 2.

### Identification of Bacteria With High 1,3-Xylanase-Secreting Ability

**Figure 3:**
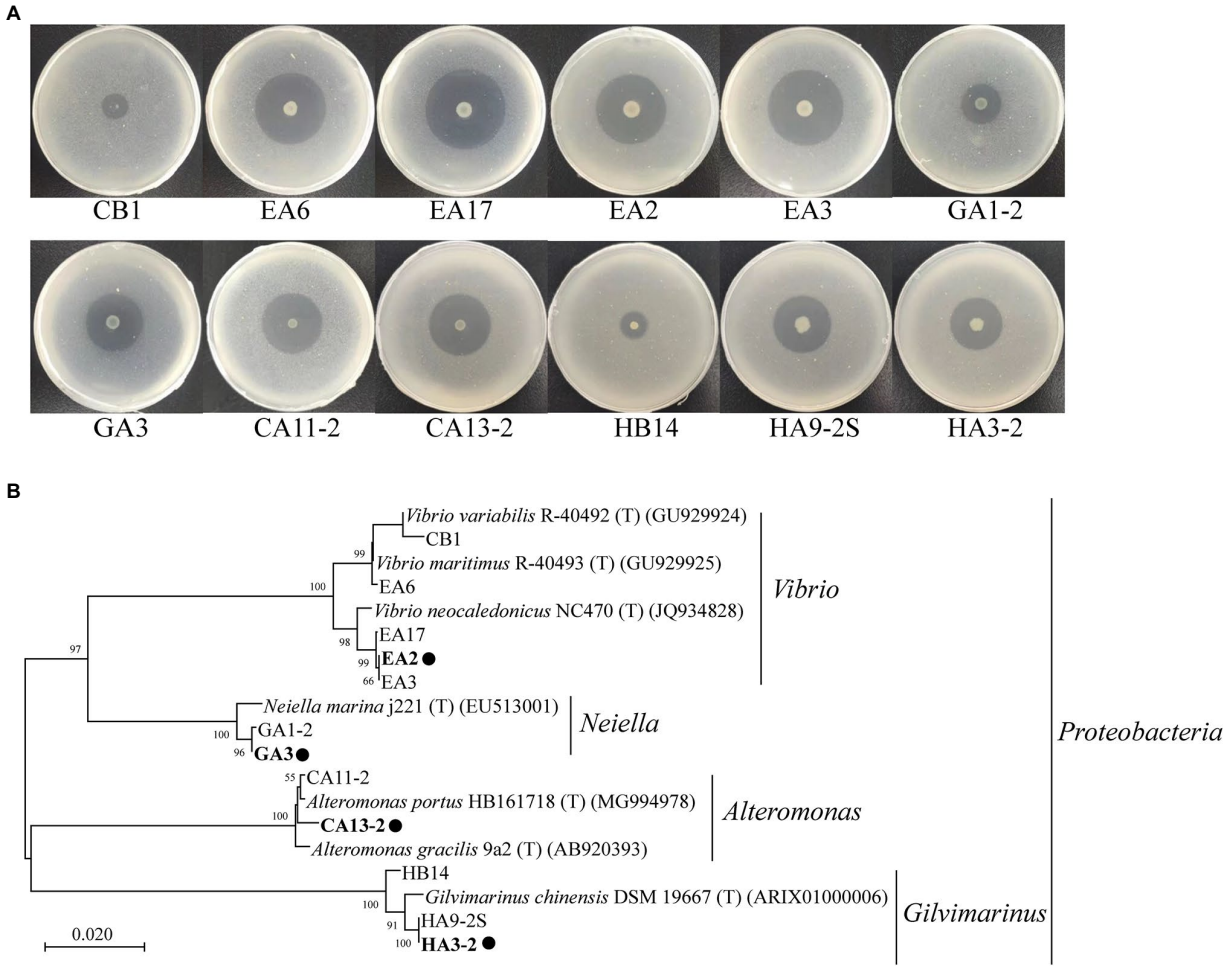
Hydrolytic zones of the isolated 1,3-xylanase-secreting bacteria cultured on plates containing 0.2% 1,3-xylan **(A)** and the neighbor-joining phylogenetic tree based on the 16S rRNA gene sequences of these strains and their closest neighbors **(B)**. These strains were cultured at 30°C for 5days for the observation of their hydrolytic zones. The neighbor-joining tree was built using the Kimura 2-parameter model (Kimura, 1980). A bootstrap test of 1,000 replicates was conducted, and values above 50% are shown. Representative strains for detailed study are marked with solid circles.

Among the culturable bacteria, 12 strains formed apparent hydrolytic zones on the plates containing 1,3-xylan (Table 3; Figure 3A), suggesting that these strains have a high 1,3-xylanase-secreting ability. A neighbor-joining tree based on the 16S rRNA gene sequences illustrated their phylogenetic relationship with different genera (Figure 3B). The 12 strains were closely related to their closest neighbors in *Vibrio* (5 of 12 strains), *Neiella* (2 of 12 strains), *Altermonas* (2 of 12 strains), and *Gilvimarinus* (3 of 12 strains), all belonging to phylum *Proteobacteria* (Table 3; Figure 3B). Also, all the reference strains are from marine sources. In these genera, only *Vibrio* strains have been reported to secrete 1,3-xylanase, including *Vibrio* sp. XY214 (Araki et al., 1999) and *Vibrio* sp. AX-4 (Araki et al., 1987). Strains in the other 3 genera are first found to have the 1,3-xylanase-secreting ability.

**Table 3:** Information of the isolated 1,3-xylanase-secreting bacteria.

Strain	Isolation sample	Screening medium	The closest neighbor	Similarity (%)	Completeness (%)
CB1	C	B	*Vibrio variabilis* R-40492 (T)	99.92	96.0
EA6	E	A	*Vibrio maritimus* R-40493 (T)	99.03	98.6
EA17	E	A	*Vibrio neocaledonicus* NC470 (T)	99.01	98.7
EA2	E	A	*Vibrio neocaledonicus* NC470 (T)	99.01	98.2
EA3	E	A	*Vibrio neocaledonicus* NC470 (T)	99.01	98.8
GA1-2	G	A	*Neiella marina* j221 (T)	98.26	98.7
GA3	G	A	*Neiella marina* j221 (T)	97.91	98.4
CA11-2	C	A	*Alteromonas portus* HB161718 (T)	99.43	96.3
CA13-2	C	A	*Alteromonas gracilis* 9a2 (T)	99.12	98.8
HB14	H	B	*Gilvimarinus chinensis* DSM 19667 (T)	96.65	98.8
HA9-2S	H	A	*Gilvimarinus chinensis* DSM 19667 (T)	98.25	98.6
HA3-2	H	A	*Gilvimarinus chinensis* DSM 19667 (T)	98.18	98.8

**Figure 4:**
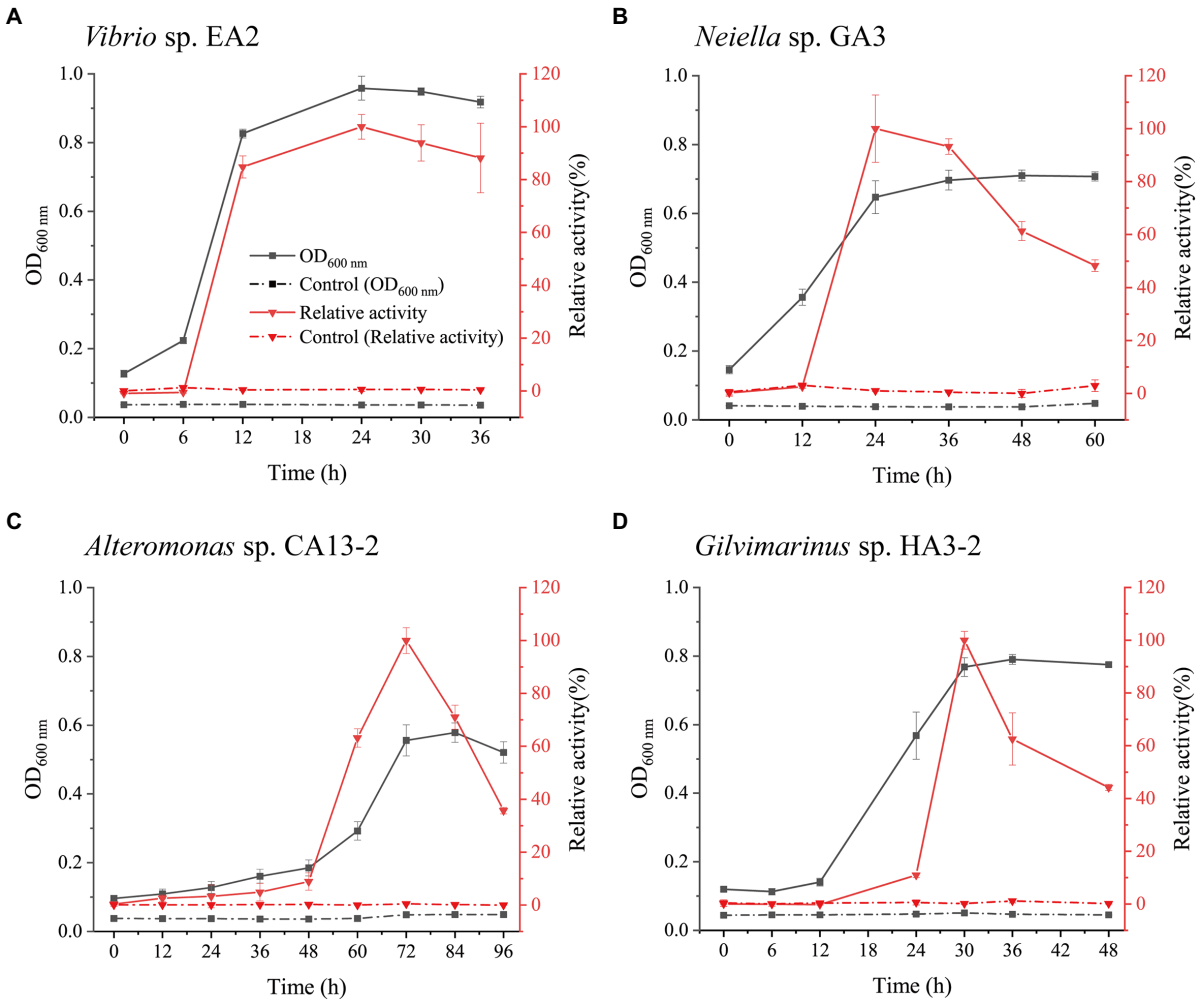
Growth and the extracellular 1,3-xylanase activities of *Vibrio* sp. EA2 **(A)**, *Neiella* sp. GA3 **(B)**, *Alteromonas* sp. CA13-2 **(C),** and *Gilvimarinus* sp. HA3-2 **(D)** cultured with 0.2% 1,3-xylan as the carbon source. The 1,3-xylanase activities were determined at 30°C in PBS (pH 7.0), and the highest activity was taken as 100%. The highest activities were 0.25±0.01U/mL (for *Vibrio* sp. EA2), 0.06±0.001U/mL (for *Neiella* sp. GA3), 0.21±0.01U/mL (for *Alteromonas* sp. CA13-2), and 0.10±0.01U/mL (for *Gilvimarinus* sp. HA3-2). Cultures without 1,3-xylan were treated as controls. The data shown in the graph are from triplicate experiments (mean±S.D.).

*Vibrio* sp. EA2, *Neiella* sp. GA3, *Alteromonas* sp. CA13-2, and *Gilvimarinus* sp. HA3-2 were chosen from the four genera as representatives for further study. With 1,3-xylan as the carbon source, we determined the growth curves of the 4 strains. Meanwhile, the extracellular 1,3-xylanase activity in the culture during bacterial growth was monitored. The results showed that these 4 strains effectively used 1,3-xylan as the carbon source for their growth (Figure 4). The extracellular 1,3-xylanase activity reached the highest level at the late-log phase for each strain (Figure 4). In addition, when cultured with *Caulerpa lentillifera*, these 4 strains also effectively decomposed intact algae for their growth (Supplementary Figure 4).

### Biochemical Characterization of the Extracellular 1,3-Xylanases

**Figure 5:**
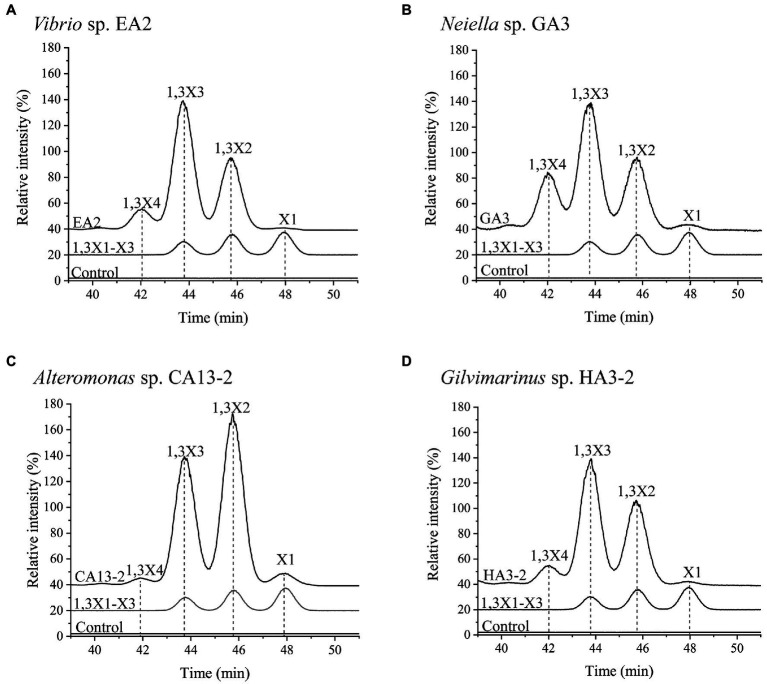
The products released from 1,3-xylan by the hydrolysis of the extracellular 1,3-xylanases secreted by *Vibrio* sp. EA2 **(A)**, *Neiella* sp. GA3 **(B)**, *Alteromonas* sp. CA13-2 **(C)**, and *Gilvimarinus* sp. HA3-2 **(D)**. Extracellular 1,3-xylanases from each strain were incubated with 1,3-xylan at 30°C for 24h. Then, the products were analyzed by HPLC-ELSD. The intensity of the 1,3-xylotriose in each chromatogram was normalized as 100%. The reaction system without 1,3-xylanases was used as the control. The data are representative of the results of triplicate experiments.

To characterize the extracellular 1,3-xylanases, culture supernatants of *Vibrio* sp. EA2, *Neiella* sp. GA3, *Alteromonas* sp. CA13-2, and *Gilvimarinus* sp. HA3-2 at the late-log phase were collected. The extracellular 1,3-xylanases produced by *Vibrio* sp. EA2 exhibited the highest activity at 40°C and pH 7.0 in 0M NaCl. After 1-h incubation at 50°C, they retained 45% activity, indicating a thermo-unstable character at 50°C. However, they showed an excellent tolerance over a wide pH range, retaining more than 80% activity at pH 5.0–10.0 (Table 4; Supplementary Figure 5). Their products released from 1,3-xylan were 1,3X2, 1,3X3 and a small amount of 1,3X4 (Figure 5A). The extracellular 1,3-xylanases produced by *Neiella* sp. GA3 exhibited the highest activity at 40°C and pH 7.0 in 0.5M NaCl. They retained 57% activity after 1-h incubation at 50°C and~90% activity over a wide pH range of 6.0–10.0 (Table 4; Supplementary Figure 6). Their products released from 1,3-xylan were 1,3X2, 1,3X3, 1,3X4, and a trace amount of xylose (Figure 5B). The extracellular 1,3-xylanases produced by *Alteromonas* sp. CA13-2 exhibited the highest activity at 30°C and pH 7.0 in 0.1M NaCl. After 1-h incubation at 40°C and 50°C, 22% and 1% activities were retained, respectively. After 24-h incubation at pH 4.0–9.0, more than 80% activity was retained. Even at pH 10.0, they retained 66% activity, indicating their alkali resistance (Table 4; Supplementary Figure 7). They acted on 1,3-xylan and released 1,3X2, 1,3X3, and a small amount of xylose and 1,3X4 (Figure 5C). The extracellular 1,3-xylanases produced by *Gilvimarinus* sp. HA3-2 exhibited the highest activity at 30°C and pH 6.0 in 0.1M NaCl. They lost almost all activity after 1-h incubation at 50°C. At pH 10.0, they retained 58% activity (Table 4; Supplementary Figure 8). Their products released from 1,3-xylan were 1,3X2 and 1,3X3 and a small amount of 1,3X4. The produced xylose was negligible (Figure 5D). Together, the extracellular 1,3-xylanases produced by these 4 strains exhibit the highest activity at mesophilic conditions and neutral pH in 0–0.5M NaCl and hydrolyze 1,3-xylan into xylooligosaccharides mainly 1,3X2 and 1,3X3. In addition, these 1,3-xylanases are thermo-unstable at 50°C, but exhibit good alkali resistance.

**Table 4:** Biochemical characterization of the extracellular 1,3-xylanases secreted by *Vibrio* sp. EA2, *Neiella* sp. GA3, *Alteromonas* sp. CA13-2, and *Gilvimarinus* sp. HA3-2^a^.

Strain	Optimum			Stability	
	Temperature (°C)	pH	NaCl concentration (M)	Temperature (°C)^b^	pH^c^
*Vibrio* sp. EA2	40	7.0	0.0	≤30°C	pH 5.0–10.0
*Neiella* sp. GA3	40	7.0	0.5	≤30°C	pH 6.0–10.0
*Alteromonas* sp. CA13-2	30	7.0	0.1	≤20°C	pH 4.0–9.0
*Gilvimarinus* sp. HA3-2	30	6.0	0.1	≤30°C	>75%, pH 5.0–6.0

a Detailed information was shown in Supplementary Figures 5–8.

b The extracellular 1,3-xylanases were incubated at from 10°C to 60°C. If the 1,3-xylanases still retained more than 80% of the enzyme activity after 1-h incubation, they were considered stable at this temperature.

c The extracellular 1,3-xylanases were incubated in the Britton-Robinson buffer with different pH values (pH 4.0–10.0) for 24h. If the 1,3-xylanases still retained more than 80% of the enzyme activity after incubation, they were considered stable at this pH.

## Discussion

Bacteria that decompose algal polysaccharides are thought to be pivotal in marine carbon cycling (Williams et al., 2013; Hehemann et al., 2014). However, for 1,3-xylan, the main structural constituent in the cell walls of some red and green algae (Iriki et al., 1960; Veluraja and Atkins, 1987), only 4 1,3-xylanase-secreting bacteria have been isolated from marine sources including *Vibrio* sp. AX-4 (Araki et al., 1987), *Pseudomonas* sp. PT-5 (Yamaura et al., 1990), *Alcaligenes* sp. XY-234 (Araki et al., 1998), and *Vibrio* sp. XY-214 (Araki et al., 1999). *Pseudomonas* sp. PT-5 was isolated from edible seaweed, nori, and the other 3 bacterial strains were isolated from the sea mud samples. Despite these studies, there has been no report on the diversity of bacteria involved in the degradation and utilization of 1,3-xylan. In this study, with the 16S rRNA gene amplicon sequencing and the culture-dependent method, the diversity of 1,3-xylan-utilizing bacteria from algae collected from Hainan, China, was investigated. After enrichment with 1,3-xylan, bacteria from phyla *Proteobacteria* and *Bacteroidetes* take a leading role in the bacterial community, consistent with the finding that *Proteobacteria* and *Bacteroidetes* are master decomposers for algal polysaccharides (Teeling et al., 2012; Barbeyron et al., 2016). In these two phyla, at least 19 families were determined, unveiling that a wide range of marine bacteria has the 1,3-xylan-utilizing ability.

In this study, we investigate the bacteria involved in 1,3-xylan degradation and utilization associated with *Caulerpa sertularioides*, *Caulerpa lentillifera*, and *Chaetomorpha* sp. Among these algae, only the bacterial diversity associated with *Caulerpa lentillifera* has been analyzed with the 16S rRNA gene amplicon sequencing (Liang et al., 2019), which showed that the bacteria are predominantly from phyla *Proteobacteria* (52.1%), *Planctomycetes* (21.1%), *Bacteroidetes* (13.5%), and *Cyanobacteria* (7.8%). Our results in this study showed that 1,3-xylan-utilizing bacteria associated with *Caulerpa lentillifera* mainly belong to phyla *Proteobacteria* (71.4%) and *Bacteroidetes* (28.1%); however, the recovered *Planctomycetes* bacteria (0.02%) are negligible and no *Cyanobacteria* bacteria are recovered from *Caulerpa lentillifera*.

Bacteria from genera *Vibrio*, *Pseudomonas,* and *Alcaligenes* have been reported to secrete 1,3-xylanases (Yamaura et al., 1990; Araki et al., 1998, 1999; Kiyohara et al., 2005). In this study, 12 bacterial strains with high 1,3-xylanase-secreting ability were isolated, which formed apparent hydrolytic zones on plates containing 1,3-xylan. Among them, bacteria belonging to genera *Neiella*, *Altermonas*, and *Gilvimarinus* are first found to secrete 1,3-xylanases. Interestingly, the 12 strains isolated in this study and their closest neighbors are all from coastal environments, where the bacterial response to phytoplankton blooms is dynamic (Teeling et al., 2012). The neighbors *Gilvimarinus chinensis* DSM 19667 and *Alteromonas portus* HB161718 have been shown to have agar- and alginate-digesting capacity, respectively (Du et al., 2009; Huang et al., 2020). Probably, the 12 strains isolated in this study also exhibit the capacity to degrade other algal polysaccharides and participate in coastal polysaccharides degradation as specific or versatile degraders, which, however, needs further investigation. Notably, only 12 of 146 (8.2%) culturable bacteria were isolated with high 1,3-xylanase-secreting ability. There are two possible reasons for this. First, there may be a considerable number of bacteria whose 1,3-xylanase-secreting capacity is too weak to form an apparent hydrolytic zone around its colony. Second, a proportion of culturable bacteria may be unable to degrade 1,3-xylan and rely on 1,3-xylooligosaccharides generated by other bacteria for growth.

1,3-xylanases play a key role in 1,3-xylan degradation and recycling in the ocean, which also exhibit vast potentials in bioenergy, pharmaceutical, and biotechnology industries (Araki et al., 1994; Maeda et al., 2012; Umemoto et al., 2012). Thus, the discovery of 1,3-xylanases from 1,3-xylanase-secreting bacteria will facilitate the utilization of algal resources. Previously reported 1,3-xylanase from *Vibrio* sp. AX-4 has the highest activity at pH 6.0–7.5 and 30°C (Araki et al., 1987; Kiyohara et al., 2005). The 1,3-xylanase from *Pseudomonas* sp. PT-5 shows its maximum activity at pH 7.5 and is stable at pH 5.5–8.0 (Yamaura et al., 1990). 1,3-xylanases from *Alcaligenes* sp. XY-234 (Araki et al., 1998) and *Vibrio* sp. XY-214 (Araki et al., 1999) have been well studied. They exhibit the highest activity at pH 7.0–7.5 and 37–40°C and are stable under 40°C and 30°C, respectively. Moreover, the 1,3-xylanases produced by *Alcaligenes* sp. XY-234 are stable at pH 6.0–10.0, showing good alkali resistance. In this study, extracellular 1,3-xylanases secreted by 4 representative strains were biochemically characterized. The 1,3-xylanases from the strains show the highest activity at pH 6.0–7.0 and 30–40°C and remain high activities at pH 8.0, suggesting their adaptation to the marine environment where these strains were isolated. These 1,3-xylanases are thermo-unstable but alkali resistance. At the corresponding optimum conditions, the activities of the 1,3-xylanases from the 4 strains are in the range of 0.07–0.53U/mL, higher than that produced by *Vibrio* sp. AX-4 (0.03U/ml; Araki et al., 1987). Moreover, the products of these enzymes to hydrolyze 1,3-xylan are mainly 1,3X2 and 1,3X3, similar to those of xylanases produced by *Vibrio* sp. AX-4 (Araki et al., 1987), *Alcaligenes* sp. XY-234 (Araki et al., 1998), and *Vibrio* sp. XY-214 (Araki et al., 1999). Thus, these 1,3-xylanases may have potential in the preparation of 1,3-xylooligosaccharides. These results lay a foundation for the discovery of 1,3-xylanases.

In summary, this study reveals the diversity of marine bacteria involved in the degradation and utilization of 1,3-xylan. Twelve strains with high 1,3-xylanase-secreting capacity were isolated, and the extracellular 1,3-xylanases secreted by 4 representative strains were biochemically characterized. These findings shed light on the ecological functions of 1,3-xylanase-secreting bacteria and their extracellular 1,3-xylanases. The research also helps in further elucidating the degradation mechanism of 1,3-xylan and discovering novel 1,3-xylanases. Detailed studies on the 1,3-xylanase-secreting bacteria and 1,3-xylanases are on the way.

## Data Availability Statement

The datasets presented in this study can be found in online repositories. The names of the repository/repositories and accession number(s) can be found at: https://www.ncbi.nlm.nih.gov/genbank/ (MZ340577, MZ340626, MZ342596, MZ342745, MZ342757, MZ342758, MZ342759, MZ340547, MZ340550, MZ342767, MZ342768, MZ342787, MZ821015, and from MZ816037 to MZ816169) and at: https://www.ncbi.nlm.nih.gov/sra/ (from SRR15511288 to SRR15511295).

## Author Contributions

FZ, X-LC, and H-NS designed and directed the research. H-NS and C-MY performed the experiments. Z-GF helped in sample collection. H-HF and PW helped in data analysis. FZ and H-NS wrote the manuscript. X-LC and Y-ZZ revised the manuscript. All authors contributed to the article and approved the submitted version.

## Funding

The work was supported by the Major Scientific and Technological Innovation Project (MSTIP) of Shandong Province (2019JZZY010817 awarded to Y-ZZ), the National Science Foundation of China (grants U2006205 and U1706207, awarded to X-LC and Y-ZZ, respectively), and Taishan Scholars Program of Shandong Province (tspd20181203 awarded to Y-ZZ).

## Conflict of Interest

The authors declare that the research was conducted in the absence of any commercial or financial relationships that could be construed as a potential conflict of interest.

## Publisher’s Note

All claims expressed in this article are solely those of the authors and do not necessarily represent those of their affiliated organizations, or those of the publisher, the editors and the reviewers. Any product that may be evaluated in this article, or claim that may be made by its manufacturer, is not guaranteed or endorsed by the publisher.

## Footnotes

^1^ http://www.cazy.org/

^2^ https://www.ezbiocloud.net/
