# Supplementary material for: Diversity of Marine 1,3-Xylan-Utilizing Bacteria and Characters of Their Extracellular 1,3-Xylanases
Source: Front Microbiol. 2021 Oct 1;12:721422. doi: 10.3389/fmicb.2021.721422 (PMC8517272; doi:10.3389/fmicb.2021.721422)
Supplement: Supplementary file 1 [file Data_Sheet_1.DOCX]

Supplementary Tables and Figures

**Supplementary Table 1** Dominant families of bacteria recovered from algal samples with 1,3-xylan in media A and B based on 16S rRNA gene amplicon sequencing.

| Isolation sample | Dominant families (relative abundance) | |
| --- | --- | --- |
|  | Medium A | Medium B |
| C | *Thalassospiraceae* (34.7%)  *Vibrionaceae* (31.0%)  *Rhodobacteraceae* (13.2%) | *Vibrionaceae* (46.3%)  *Rhodobacteraceae* (30.6%)  *Flavobacteriaceae* (10.5%) |
| E | *Flavobacteriaceae* (68.1%)  *Rhodobacteraceae* (15.8%) | *Flavobacteriaceae* (59.7%)  *Rhodobacteraceae* (22.4%) |
| G | *Rhodobacteraceae* (30.5%)  *Rhizobiaceae* (18.8%)  *Nitrincolaceae* (12.4%) | *Rhodobacteraceae* (21.6%)  *Rhizobiaceae* (15.4%)  *Crocinitomicaceae* (14.0%) |
| H | *Rhodobacteraceae* (39.3%)  *Rhizobiaceae* (20.0%)  *Alteromonadaceae* (15.3%) | *Rhodobacteraceae* (64.1%)  *Crocinitomicaceae* (11.5%)  *Flavobacteriaceae* (5.5%) |

**Supplementary Table 2** Phylogenetic classification and relative abundance of culturable bacteria isolated from the enrichment cultures of the algal samples ^a^.

| Phylogenetic classification | | | Sample | | | | | | | |
| --- | --- | --- | --- | --- | --- | --- | --- | --- | --- | --- |
|  |  |  | C | | E | | G | | H | |
|  |  |  | Medium | | | | | | | |
| Phylum  (No./Abundance) | Family  (No./Abundance) | Genus  (No.) | A | B | A | B | A | B | A | B |
| *Proteobacteria*  (128 / 87.7%) | *Vibrionaceae* (24/16.4%) | *Vibrio* (24) | CA1, CA5, CA7, CA13-1, CA14, CA15-1 | CB1, CB3, CB4, CB6-2, CB8, CB9-1 | EA2, EA3, EA6, EA11, EA17 | 0 | GA15-1 | 0 | 0 | HB5, HB6-1, HB6-2, HB7-1, HB7-3, HB7-4 |
|  | *Rhodobacteraceae* (19/13.0%) | *Pseudoruegeri*a (8) | 0 | CB5-1, CB5-2, CB5-3, CB6-1, CB9-2, CB15, CB17 | EA1 | 0 | 0 | 0 | 0 | 0 |
|  |  | *Ruegeria* (6) | 0 | 0 | EA10 | EB6, EB8 | 0 | 0 | HA3-1, HA6-1, HA6-2 | 0 |
|  |  | *Salipiger* (1) | 0 | 0 | 0 | 0 | GA2-2 | 0 | 0 | 0 |
|  |  | *Pseudooceanicola* (2) | 0 | CB13 | 0 | 0 | 0 | GB1 | 0 | 0 |
|  |  | *Celeribacter* (2) | 0 | 0 | 0 | 0 | 0 | GB3-2, GB11-2 | 0 | 0 |
|  | *Phyllobacteriaceae* (17/11.6%) | *Nitratireductor* (16) | 0 | 0 | EA4-1, EA8, EA14, EA18 | EB10, EB15 | GA17 | GB3-1, GB5, GB6 | HA5-1, HA5-3, HA7, HA9-1, HA11, HA12-1 | 0 |
|  |  | *Mesorhizobium* (1) | 0 | 0 | 0 | EB3 | 0 | 0 | 0 | 0 |
|  | *Cellvibrionaceae* (14/9.6%) | *Gilvimarinus* (14) | 0 | 0 | EA4-2, EA12, EA13, EA16 | 0 | 0 | 0 | HA3-2, HA9-2S, HA9-3, HA10 | HB8, HB9-1, HB13, **HB14**, HB15, HB17 |
|  | *Alteromonadaceae* (9/6.2%) | *Alteromonas* (7) | CA11-2, CA13-2 | CB10, CB14 | 0 | 0 | 0 | 0 | HA1, HA12-2, HA16 | 0 |
|  |  | *Marisediminitalea* (1) | 0 | 0 | 0 | 0 | 0 | 0 | **HA8** | 0 |
|  |  | *Ningiella* (1) | 0 | 0 | 0 | 0 | 0 | GB2 | 0 | 0 |
|  | *Alteromonadales* (9/6.2%) | *Neiella* (9) | 0 | 0 | 0 | 0 | GA1-2, GA3, GA4-2, GA5, GA9, GA11, GA12, GA13, GA14-1 | 0 | 0 | 0 |
|  | *Pseudoalteromonadaceae* (7/4.8%) | *Pseudoalteromonas* (7) | CA4-1 | 0 | 0 | 0 | GA7, GA8, GA15-2, GA16 | 0 | HA5-2 | HB1 |
|  | *Enterobacteriaceae* (5/3.4%) | *Enterobacter* (5) | 0 | CB1-2, CB12-1 | 0 | EB7 | 0 | GB7-1, GB16 | 0 | 0 |
|  | *Pseudomonadaceae* (5/3.4%) | *Pseudomonas* (5) | 0 | 0 | 0 | 0 | 0 | GB10-1, GB12, GB13, GB15 | HA4 | 0 |
|  | *Marinobacteraceae* (5/3.4%) | *Marinobacter* (5) | 0 | 0 | EA5 | 0 | 0 | 0 | 0 | HB2, HB4, HB12-1, HB12-2 |
|  | *Halomonadaceae* (4/2.7%) | *Halomonas* (4) | 0 | 0 | 0 | EB4, EB9-1, EB9-2, EB11 | 0 | 0 | 0 | 0 |
|  | *Thalassospiraceae* (3/2.1%) | *Thalassospira* (3) | CA8, CA16-1 | 0 | 0 | 0 | 0 | 0 | HA17 | 0 |
|  | *Idiomarinaceae* (2/1.3%) | *Pseudidiomarina* (2) | 0 | 0 | 0 | 0 | GA1-1 | GB9 | 0 | 0 |
|  | *Stappiaceae* (1/0.7%) | *Roseibium* (1) | CA9 | 0 | 0 | 0 | 0 | 0 | 0 | 0 |
|  | *Kangiellaceae* (1/0/7%) | *Kangiella* (1) | 0 | 0 | 0 | EB13 | 0 | 0 | 0 | 0 |
|  | *Erythrobacteraceae* (1/0.7%) | *Erythrobacter* (1) | 0 | 0 | 0 | 0 | 0 | GB14 | 0 | 0 |
|  | *Oceanospirillaceae* (1/0.7%) | *Marinobacterium* (1) | 0 | 0 | 0 | 0 | 0 | 0 | 0 | HB3 |
|  | *Hyphomonadaceae* (1/0.7%) | *Maricaulis* (1) | 0 | 0 | 0 | 0 | 0 | 0 | HA15 | 0 |
| *Bacteroidetes*  (17 / 11.6%) | *Crocinitomicaceae* (6/4.1%) | *Wandonia* (6) | 0 | 0 | 0 | 0 | 0 | GB7-2, GB10-2, GB11-1 | HA9-1S, HA9-2 | HB9-2 |
|  | *Cyclobacteriaceae* (5/3.4%) | *Algoriphagus* (3) | 0 | 0 | EA9-2 | EB5, EB12 | 0 | 0 | 0 | 0 |
|  |  | *Cyclobacterium* (2) | 0 | 0 | 0 | 0 | GA10-1, GA10-2 | 0 | 0 | 0 |
|  | *Flavobacteriaceae* (3/2.1%) | *Tenacibaculum* (2) | 0 | 0 | EA9-1, EA9-3 | 0 | 0 | 0 | 0 | 0 |
|  |  | *Mesonia* (1) | 0 | 0 | 0 | 0 | GA4-1 | 0 | 0 | 0 |
|  | *Prolixibacteraceae* (1/0.7%) | *Sunxiuqinia* (1) | 0 | 0 | 0 | 0 | 0 | GB4 | 0 | 0 |
|  | *Roseivirgaceae* (2/1.3%) | *Roseivirga* (2) | 0 | 0 | 0 | 0 | 0 | GB8 | HA13 | 0 |
| *Actinobacteria*  (1 / 0.7%) | *Microbacteriaceae* (1/0.7%) | *Microbacterium* (1) | 0 | 0 | 0 | EB2 | 0 | 0 | 0 | 0 |
| Total No. | | 146 | 12 | 18 | 19 | 14 | 20 | 20 | 24 | 19 |

^a^ Strains shown with red and bold font are potential novel species.





**Supplementary Figure 1** HPLC analysis of the prepared 1,3-xylobiose (1,3X2) and 1,3-xylotriose (1,3X3). 1,3X2 and 1,3X3 were collected from the hydrolysis products of 1,3-xylanase XYL4 on 1,3-xylan and compared to the standard 1,4-xylooligosaccharides (1,4X1-X6).


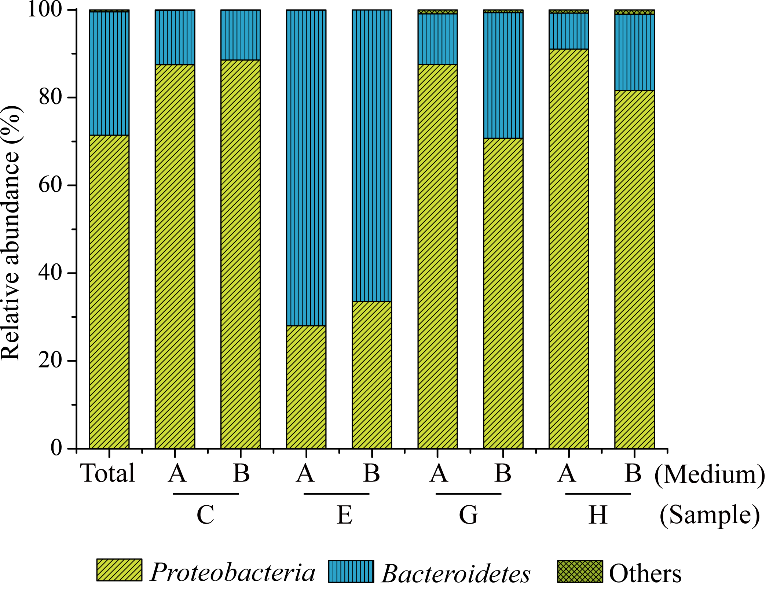


**Supplementary Figure 2** Relative abundance of recovered bacteria from algal samples with 1,3-xylan at the phylum level based on 16S rRNA gene amplicon sequencing.


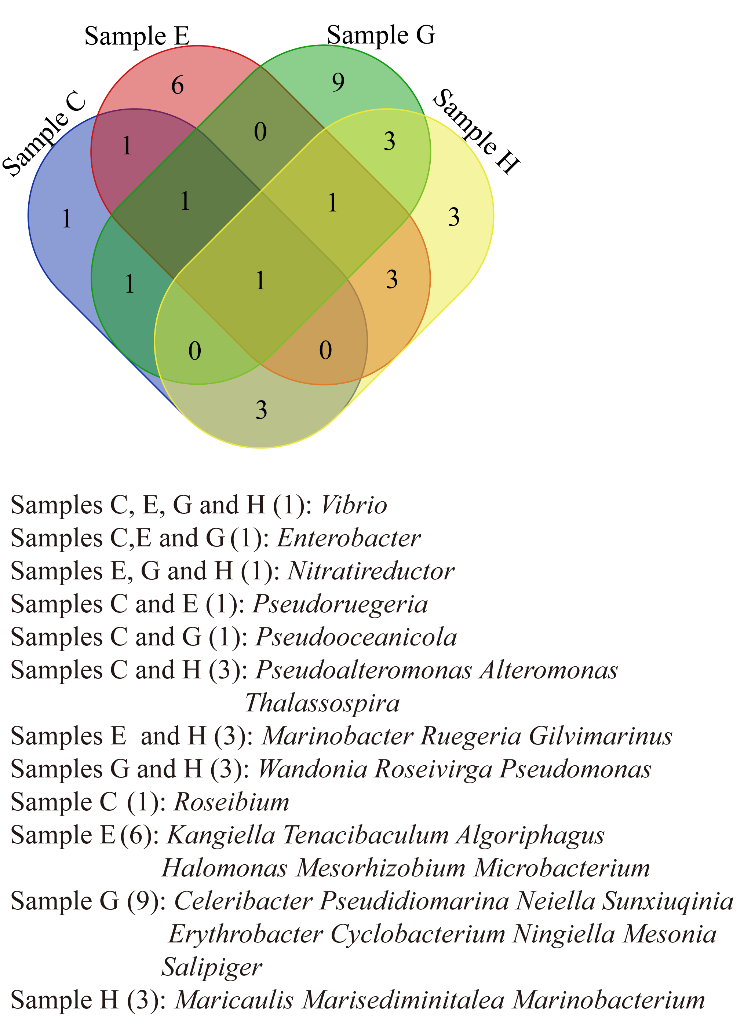


**Supplementary Figure 3** A Venn diagram analysis of the isolated genera from the enrichment cultures of algal samples.


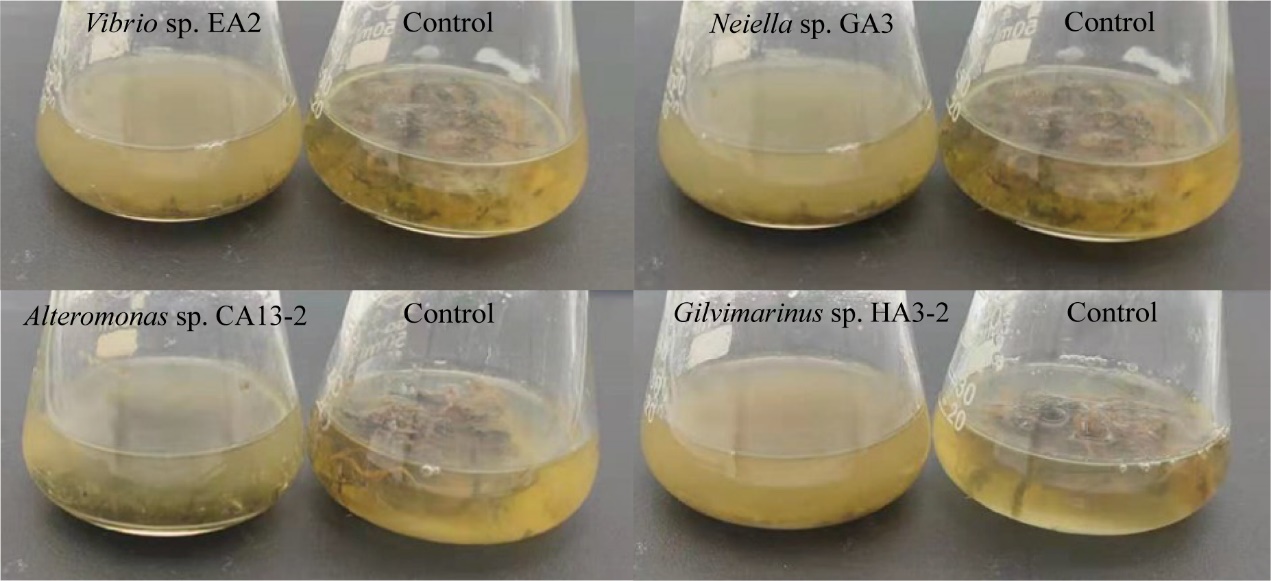


**Supplementary Figure 4** Growth of *Vibrio* sp. EA2, *Neiella* sp. GA3, *Alteromonas* sp. CA13-2 and *Gilvimarinus* sp. HA3-2 cultured with 1.0% dry *Caulerpa lentillifera.* These strains were aerobically cultured at 30°C and 180 rpm for 2 days. Cultures without bacterial inoculation were used as the control. The figure shows a representative of the results of triplicate experiments.


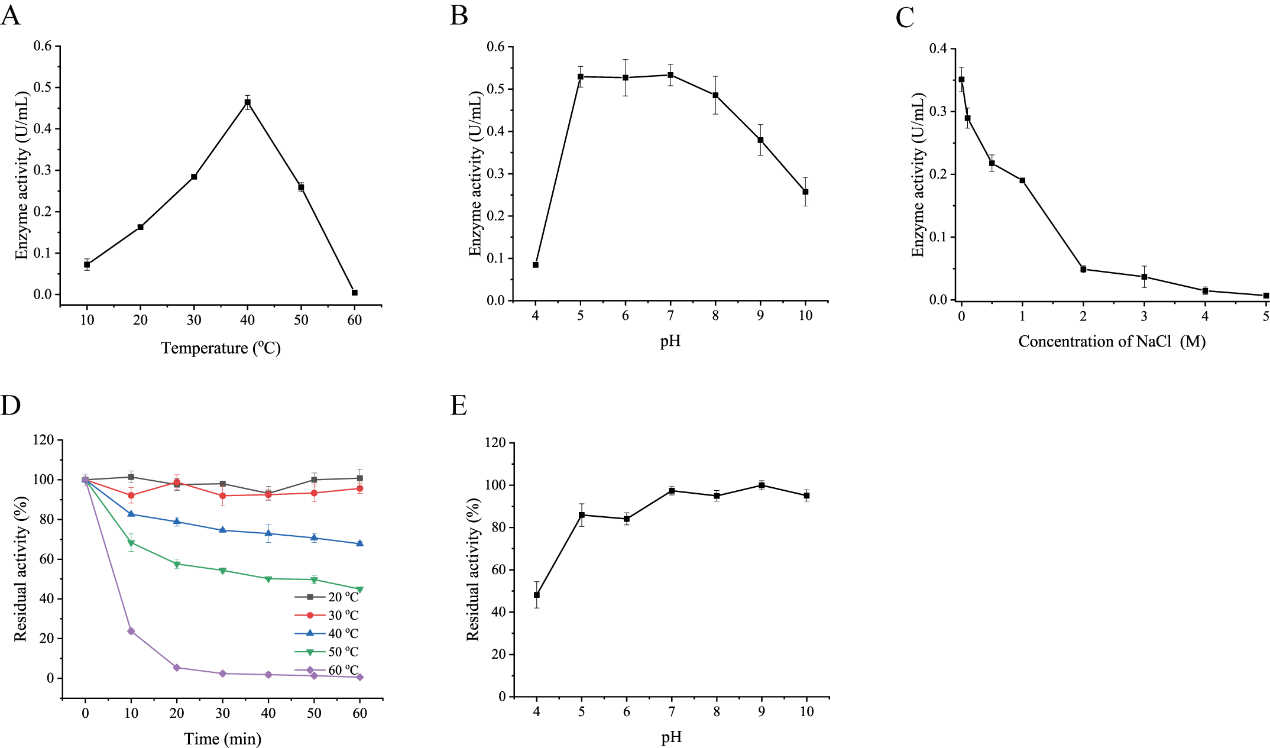


**Supplementary Figure 5** Biochemical characterization of the extracellular 1,3-xylanases secreted by *Vibiro* sp. EA2. (A) Effect of temperature on the activity of the 1,3-xylanases. (B) Effect of pH on the activity of the 1,3-xylanases. (C) Effect of NaCl concentration on the activity of the 1,3-xylanases. (D) Effect of temperature on the stability of the 1,3-xylanases. The 1,3-xylanases were incubated at 20-60°C for different time periods. The residual activity was determined at 40°C and pH 7.0 in PBS. The enzyme activity of the 1,3-xylanases incubated at 4°C (0.48 ± 0.02 U/mL) was taken as 100%. (E) Effect of pH on the stability of the 1,3-xylanases. The 1,3-xylanases were incubated in the Britton-Robinson buffer from pH 4.0 to 10.0 for 24 h. The residual activity was determined at 40°C. The enzyme activities of the 1,3-xylanases without preincubation at the corresponding pH were taken as 100%. The data shown in the graph are from triplicate experiments (mean ± S.D.).


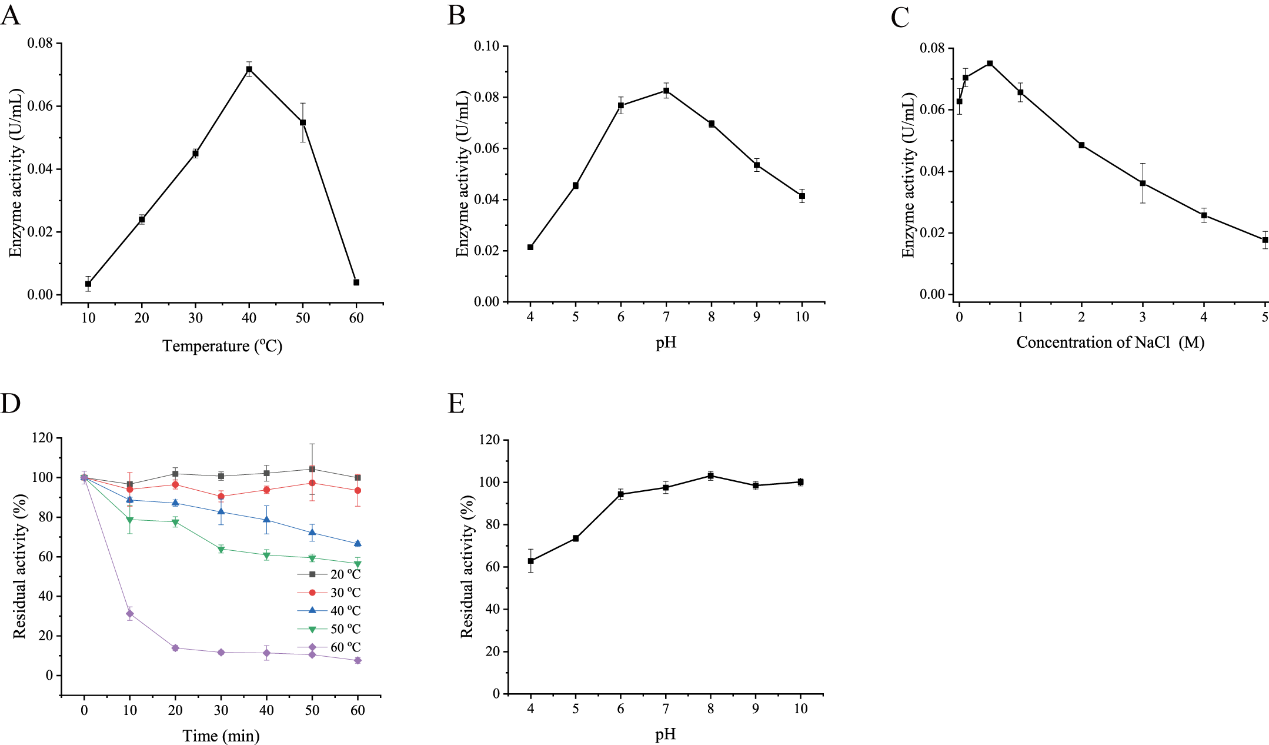


**Supplementary Figure 6** Biochemical characterization of the extracellular 1,3-xylanases secreted by *Neiella* sp. GA3. (A) Effect of temperature on the activity of the 1,3-xylanases. (B) Effect of pH on the activity of the 1,3-xylanases. (C) Effect of NaCl concentration on the activity of the 1,3-xylanases. (D) Effect of temperature on the stability of the 1,3-xylanases. The 1,3-xylanases were incubated at 20-60°C for different time periods. The residual activity was determined at 40°C and pH 7.0 in PBS. The enzyme activity of the 1,3-xylanases incubated at 4°C (0.08 ± 0.003 U/mL) was taken as 100%. (E) Effect of pH on the stability of the 1,3-xylanases. The 1,3-xylanases were incubated in the Britton-Robinson buffer from pH 4.0 to 10.0 for 24 h. The residual activity was determined at 40°C. The enzyme activities of the 1,3-xylanases without preincubation at the corresponding pH were taken as 100%. The data shown in the graph are from triplicate experiments (mean ± S.D.).


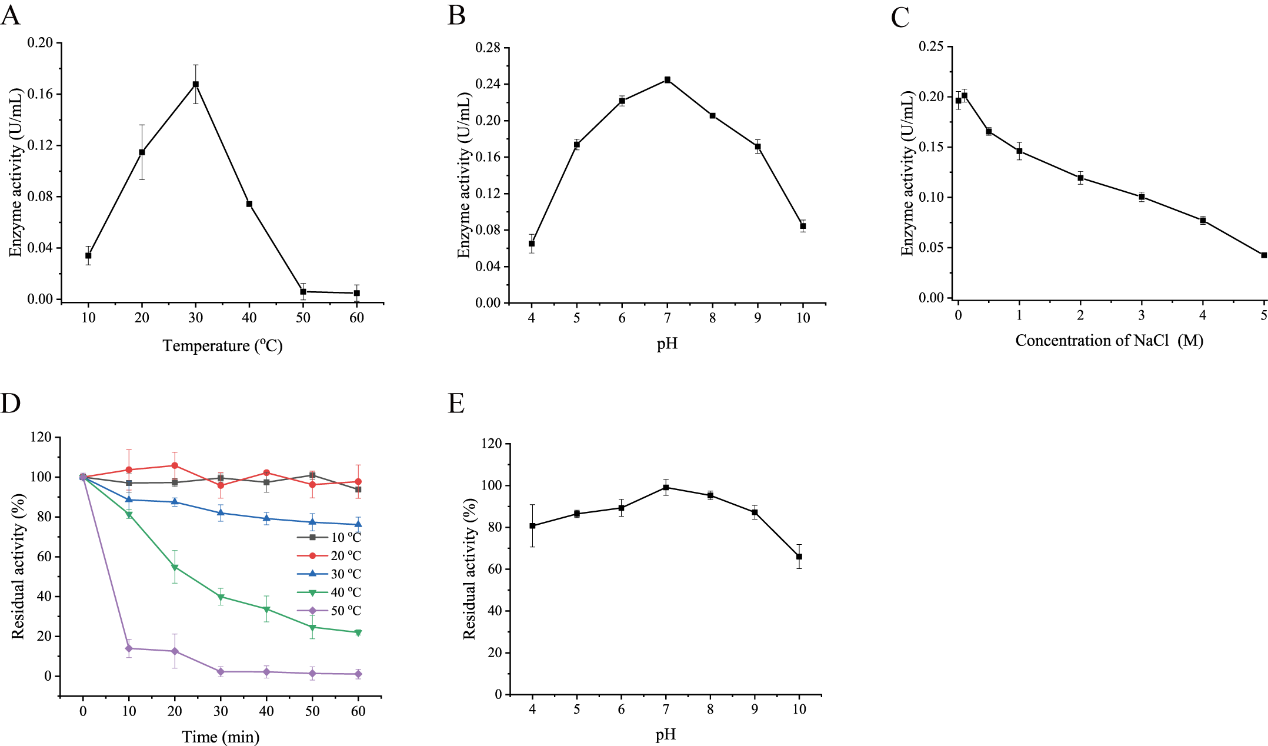


**Supplementary Figure 7** Biochemical characterization of the extracellular 1,3-xylanases secreted by *Alteromonas* sp. CA13-2. (A) Effect of temperature on the activity of the 1,3-xylanases. (B) Effect of pH on the activity of the 1,3-xylanases. (C) Effect of NaCl concentration on the activity of the 1,3-xylanases. (D) Effect of temperature on the stability of the 1,3-xylanases. The 1,3-xylanases were incubated at 10-50°C for different time periods. The residual activity was determined at 30°C and pH 7.0 in PBS. The enzyme activity of the 1,3-xylanases incubated at 4°C (0.25 ± 0.01 U/mL) was taken as 100%. (E) Effect of pH on the stability of the 1,3-xylanases. The 1,3-xylanases were incubated in the Britton-Robinson buffer from pH 4.0 to 10.0 for 24 h. The residual activity was determined at 30°C. The enzyme activities of the 1,3-xylanases without preincubation at the corresponding pH were taken as 100%. The data shown in the graph are from triplicate experiments (mean ± S.D.).


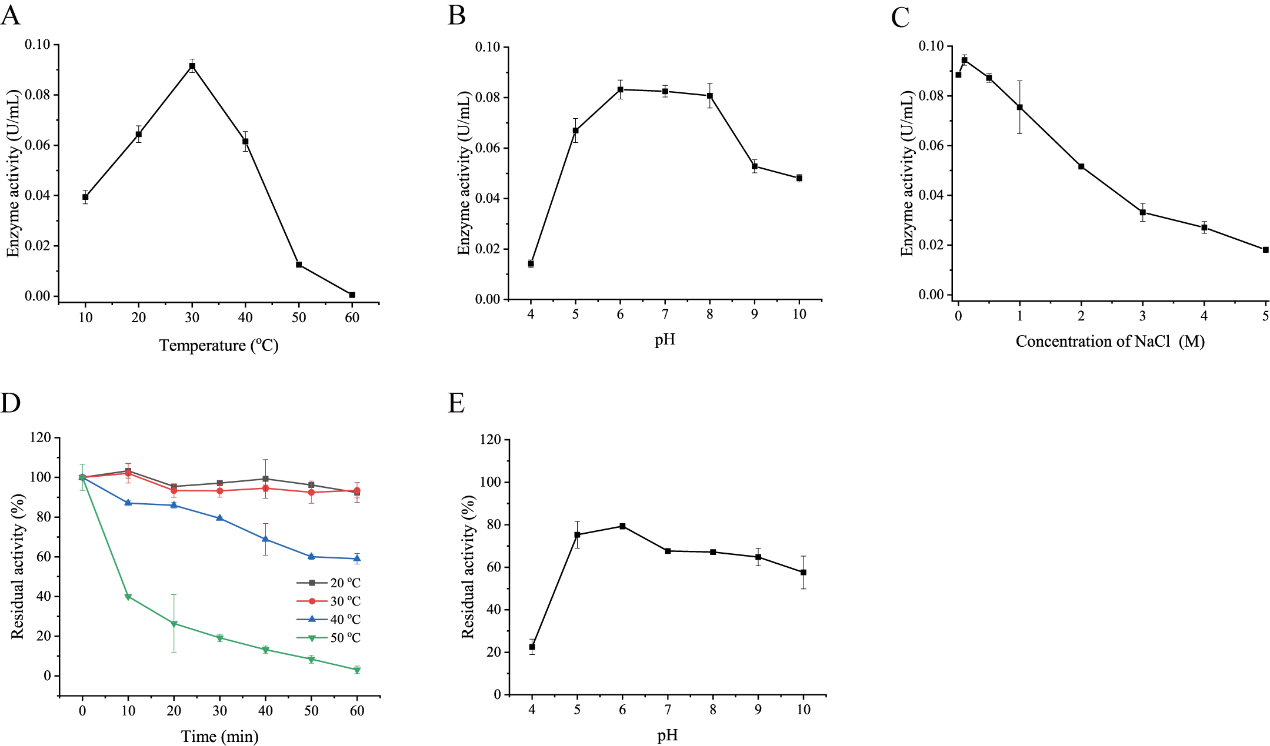


**Supplementary Figure 8** Biochemical characterization of the extracellular 1,3-xylanases secreted by *Gilvimarinus* sp. HA3-2. (A) Effect of temperature on the activity of the 1,3-xylanases. (B) Effect of pH on the activity of the 1,3-xylanases. (C) Effect of NaCl concentration on the activity of the 1,3-xylanases. (D) Effect of temperature on the stability of the 1,3-xylanases. The 1,3-xylanases were incubated at 20-50°C for different time periods. The residual activity was determined at 30°C and pH 6.0 in PBS. The enzyme activity of the 1,3-xylanases incubated at 4°C (0.08 ± 0.007 U/mL) was taken as 100%. (E) Effect of pH on the stability of the 1,3-xylanases. The 1,3-xylanases were incubated in the Britton-Robinson buffer from pH 4.0 to 10.0 for 24 h. The residual activity was determined at 30°C. The enzyme activities of the 1,3-xylanases without preincubation at the corresponding pH were taken as 100%. The data shown in the graph are from triplicate experiments (mean ± S.D.).
